# A novel oppositional binary crow search algorithm with optimal machine learning based postpartum hemorrhage prediction model

**DOI:** 10.1186/s12884-022-04775-z

**Published:** 2022-07-13

**Authors:** Sujatha Krishnamoorthy, Yihang Liu, Kun Liu

**Affiliations:** 1 Department of Computer Science, Wenzhou-Kean University, WENZHOU KEAN UNIVERSITY, 88 UNIVE, Wenzhou, 325006 Zhejiang China; 2 College of Bioscience and Bioengineering, Hebei University of Economics and Business, Shijiazhuang, 050061 Hebei China

**Keywords:** Postpartum hemorrhage, Predictive model, Machine learning, Metaheuristics, Feature Selection, Classification

## Abstract

Postpartum hemorrhage (PPH) is an obstetric emergency instigated by excessive blood loss which occurs frequently after the delivery. The PPH can result in volume depletion, hypovolemic shock, and anemia. This is particular condition is considered a major cause of maternal deaths around the globe. Presently, physicians utilize visual examination for calculating blood and fluid loss during delivery. Since the classical methods depend on expert knowledge and are inaccurate, automated machine learning based PPH diagnosis models are essential. In regard to this aspect, this study introduces an efficient oppositional binary crow search algorithm (OBCSA) with an optimal stacked auto encoder (OSAE) model, called OBCSA-OSAE for PPH prediction. The goal of the proposed OBCSA-OSAE technique is to detect and classify the presence or absence of PPH. The OBCSA-OSAE technique involves the design of OBCSA based feature selection (FS) methods to elect an optimum feature subset. Additionally, the OSAE based classification model is developed to include an effective parameter adjustment process utilizing Equilibrium Optimizer (EO). The performance validation of the OBCSA-OSAE technique is performed using the benchmark dataset. The experimental values pointed out the benefits of the OBCSA-OSAE approach in recent methods.

## Introduction

Postpartum hemorrhage (PPH) is determined as blood loss more that >500 mL within 24 hours after vaginal birth, and is a major reason for pregnancy morbidity, both in the US and world-wide [1]. The severity and incidence of PPH and the morbidity connected to blood product transfusion are increased [2]. The higher occurrences of PPH in the developing world is because of the lack of diagnosis approaches and medication utilized in the active management of the third stage. The implementation and development of quality initiatives have made aggressive supervision and early recognition of PPH with a reduction in disseminated intravascular coagulopathy, blood product transfusion, renal failure, and respiratory distress syndrome [3]. Most priorities in the PPH security bundles are identification of patients at increased risk of hemorrhage based on intrapartum and antenatal risk and the assessment of hemorrhage risk factors [4].

Existing approaches for forecasting postpartum hemorrhage depend on a risk stratification method. Enhanced prediction capacity is attained using conventional machine learning (ML) and statistical approaches [5]. Most recent developments in ML method applies modern computer-driven algorithms intended to identify pattern from data have received more interest due to their better prediction capability. Mainly though defining hospital readmission and intensive care unit admission than statistical methods. While these advanced methodologies have not been broadly tested in the obstetric field. The risk related factors attributed to PPH have been widely studied on the basis of traditional statistical models [6]. Generally, the Lasso regression model, or Logistic regression model has been utilized for predicting the risk of PPH.

Particularly, logistic or lasso regression model shows better discrimination capability [7]. According to maternal medical history and clinical characteristics, a risk score is utilized for PPH prediction. Even though the traditional predictive models are shown to be efficient, the predictive accuracy isn’t satisfied: only 60% of women having higher PPH risks are recognized, while another 40% of women who had PPH weren’t recognized at an earlier stage [8]. The benefits of ML method involve the capacity of processing non-additive relationships and integrating complex relations among other aspects which don’t require pre-determination [9]. For this reason, it is possible that ML methods are capable of precisely identifying women at the maximum risk of postpartum hemorrhage, and enhancing medical results and obstetric decision making [10].

This study develops an op positional binary crow search algorithm (OBCSA) with an optimal stacked auto encoder (OSAE) model known as OBCSA-OSAE for PPH prediction. The OBCSA-OSAE technique performs the detection and classification process of PPH. Moreover,the OBCSA-OSAE technique involves the design of OBCSA based feature selection (FS) technique for optimum feature subsets. Additionally, the OSAE based classification model is developed to include an effective parameter adjustment process using Equilibrium Optimizer (EO). The experimental outcomes highlighted that the OBCSA-OSAE technique has depicted the other techniques in terms of different evaluation parameters. The method helps to classify the PPH with classification and with good feature selection techniques. Hence, the CSA helps in the optimization algorithm. This classification result will help the doctor to give the patient an early alert and also the counseling session about them to get them prepared mentally and physically if that just happened. The whole paper is structured with an introduction and the related work in the beginning and followed by the proposed OBCSA opposing binary crow search Algorithm, process, and validation step of the model. Results are compared with the existing model, and finally, the discussion and conclusion are presented at the end of the paper with the focused results.

## Related works

Venkatesh et al. [11] employed LR method with and without lasso regularization (lasso regression) as the 2 statistical approaches, and XGBoost and RF as the 2 ML methods for predicting postpartum hemorrhage. Model accuracy was evaluated using calibration, decision curves, and C statistics (viz., concordance index). Kumar et al. [12] developed an automation method with wearable devices for preventing PPH in pregnant women. The e device evaluate parameters such as perspiration rate, temperature, pulse rate, and blood pressure. Fuzzy neural method-based rules are utilized for each parameter in predicting the risks of PPH, and for measuring the accuracy of method to reduce morbidity and mortality rates. Wu et al. [13] aim to construct a nomogram integrating clinical and radiomic features of a placenta to forecast the risks of PPH occurring in a caesarian delivery (CD). Radiomic features are selected according to their correlation with EBL. clinico-radiomic, Radiomic, radiological, clinical, and clinico-radiological methods are constructed for predicting the risks of PPH for all patients. The method with a better predictive accuracy was authenticated with its clinical application, discrimination ability, and calibration curve. Betts et al. [14] intended to forecast the risks of general maternal postpartum complication that requires inpatient care. A gradient boosting tree is utilized by 5-fold cross-validation for comparing method accuracy. The better performing methods for all the outcomes are measured later in the independent data validation with the AUC-ROC method.

Man [15] intended to apply the ML classifier methods for better PPH risk prediction. Real datasets were integrated and extracted from EHRS using twelve parameters that are considered to be very appropriate to PPH. The 6 ML methods involving LR, DT, RF, KNN, SVM, and ANN have been compared and tested based on their prediction performance and another matrix such as recall and precision. The RF method is believed to be the optimal method with 89% accuracy. Kumar et al. [16] focused on the detection and symptoms of postpartum hypothermia and hemorrhage. These wearable devices provide a life-saving product which is comfortable, affordable, and easy to use. Also, they help to alleviate other healthcare problems faced by women during and after child birth. At first, a lower cost prototype method was constructed that has sensor nodes that measure and record blood pressure, body temperature, perspiration, heart, and pulse rate. The overall purpose of these devices serve to provide efficient and accurate results to assist in the decline of morbidity rate and maternal issues.

Hochman et al. [17] validated and developed an ML based PPD predictive method using EHR data, and recognized new PPD predictor. PPD was determined by the novel diagnoses of any antidepressant prescription/depressive episode within the first postpartum year. A gradient boosting DT method was employed for clinical, obstetric features and EHR derived socio-demographic. The ML based methods that integrate HER derived predictors can increase symptoms-based screening practices by recognizing the higher risk population as a fundamental necessity for preventive interference before the onset of PPD. Yang et al. [18] adapted a univariate LR method for selecting the important features (P < 0.01). Then, they trained numerous ANN and binary LR methods for predicting postpartum hemorrhaging the NN method includes RBF, MLP, and BP. To identify and compare the precise networks, they utilized the ROC curve and the confusion matrix.

Zheutlin et al. [19] proposed a new risk assessment method and relate its performance to those employed in present practice. With a large set of potential identified and risk factor removed from EMR that was accessible previous to delivery, they trained a gradient boosting method from the subset of cohorts. In held-out test samples, they related performances of this method to three medical risk tools and one formerly published method. The result suggests that this method is an outstanding candidate for prospective assessment and can eventually decrease PPH mortality and morbidity due to earlier detection alongside prevention measures.

## The proposed OBCSA-OSAE model

**Fig. 1:**
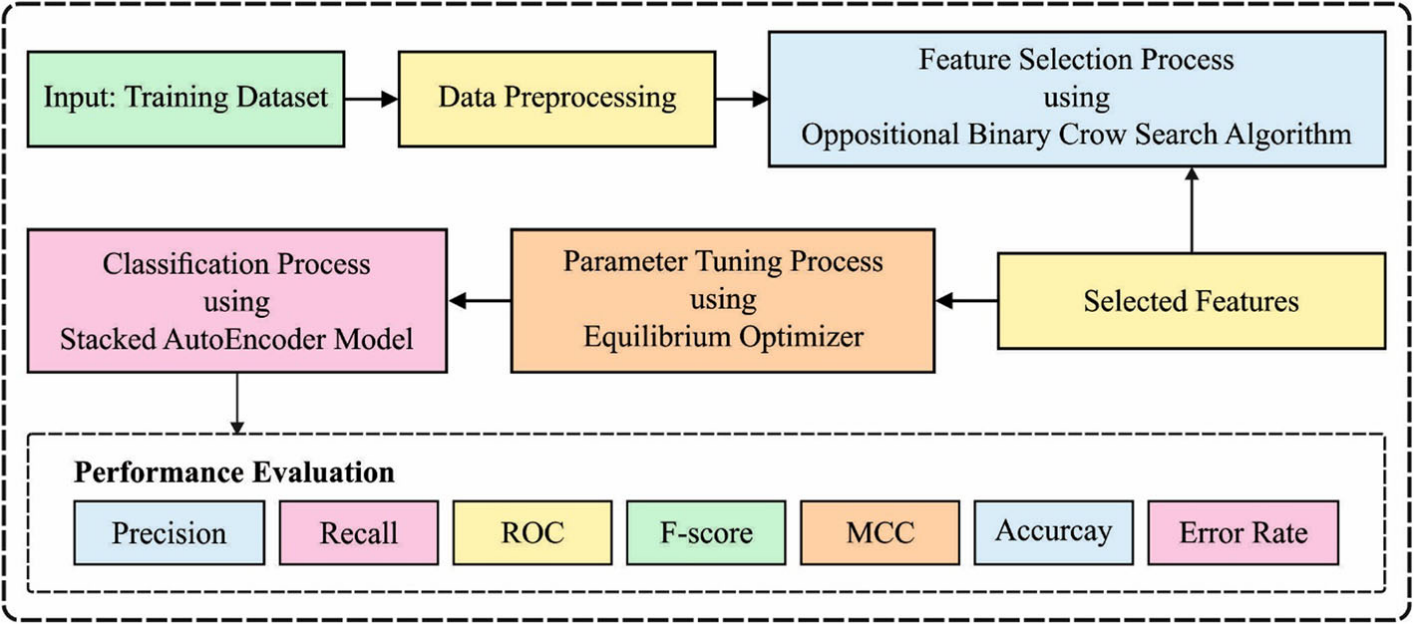
Overall process of OBCSA-OSAE model

In this study, an effective OBCSA-OSAE technique is established for the detection and classification of PPH. The proposed OBCSA-OSAE technique incorporated two major stages, choice of features and classification. Principally, the OBCSA technique is presented for optimal selection of a subset of features. Followed by the EO algorithm to derive the optimally choose parameters involved in the SAE model. Lastly, the SAE model is used for PPH classification. Figure 1 illustrates the general process of the OBCSA-OSAE model.

### Algorithmic design of OBCSA-FS technique

In the initial stage, the OBCSA-FS technique is designed to choose an optimal subset of features. The OBCSA algorithm is designed for the integration of oppositional based learning (OBL) concept alongside BCSA. The Crow Search Algorithm is one of the latest evolutionary algorithms developed by Askarzadeh [20], which was stimulated by the social habits of crow through a search procedure that mimics their behavior in the wild. The concept of CSA was inspired by the way that these animals hide food in a given place and retrieve it at a later time. Mathematically, a flock of crows is represented as *n*_*c*_, and in the search space, the position of crow *i* at iteration *t* is $x_{i}^{t} $. In CSA, the hidden location of food can be remembered by the crow *i*The procedure of upgrading the location of the crow thieves(crows who want to steal other crow food) was performed by

1 $$\begin{array}{@{}rcl@{}} x_{i}^{t+1}=x_{i}^{t}+\tau\times fl\times\left(M_{j}^{t}-x_{i}^{t}\right),\;i=1,2,\;\dots n_{c}, \end{array} $$

Whereas *fl* denotes the flight length and *τ* signifies an arbitrary value in the range of zero and one, M is a fitness function is used to evaluate each crow, and its value is put as an initial memory value for the updation.

The next state of the problem is that crow *j*, the owner of the food, knows that the crow *i* is observing him and following him, hence the crew owner would deceive crow *i* by going to any other location in the search space. In CSA, the location of crow *i* is upgraded by an arbitrary location, and the accurate state can be defined as:

2 $$\begin{array}{@{}rcl@{}} x_{i}^{t+1}= \left(\begin{array}{cc} x_{i}^{t}+\tau\times fl\times\left(M_{j}^{t}-x_{i}^{t}\right),&if\;\theta\geq AP\\ random\;position,&otherwise \end{array} \right. \end{array} $$

Whereas *θ* represents the arbitrary value from the range of [0, 1] and AP implies the probability of awareness. The CSA was adapted and is utilized in FS by suggesting a binary search model [21]. In the BCSA, the search space was developed based on the Boolean lattice of *n* dimension and the essential solution is upgraded through the corners of a hypercube, different from the typical CSA where solution is upgraded from the continuous spaces. The binary vector has been utilized for FS, whereas one equivalent to either the feature that chosen for creating the novel datasets, and or else 0. The idea of OBL has been employed the *fl* variable from the BCSA to prevent trapping from the local optimal and for enhancing the quality of resultant solution by attaining a balance among exploration as well as exploitation and obtain effective solution. The variable *fl* is initiated in the BCSA according to the OBL instead of utilizing an arbitrary initiation which might be far from the optimum global solutions and is made by:

3 $$ x=a+b-x,\;  $$

Whereas $\overline x $ represent the opposite number and *x*∈*R* denotes a real number determined on range of *x*∈[*a*,*b*]. While *a*=0 and *b*=1 Eq. (3) becomes

4 $$ x=1-x,  $$

While there is a point *P*(*x*_1_, *x*_2_, ……*x*_*n*_) in *n* dimension coordinate and *x*_1_,*x*_2_,………,*x*_*n*_∈*R* later, the opposite point $\overline P $ is determined as its coordinates $\overline {x_{1}},\overline {x_{2}},\dots,\overline {x_{n}} $:

5 $$ \overline{x_{i}}=a_{i}+b_{i}-x_{i}\;i=1,\dots\dots\dots,\;n\;\;  $$

In such cases, have 2 values, *x* represent initial arbitrary value in [*a*, *b*] and $\overline x $ denotes the opposite values of *x*. They calculate *f*(*x*)& $f(\overline x) $ in all the iterations of OBCSA, later, employ on the evaluation function *g* if $g(f(x))\geq g(f(\overline x)) $ select *x* or else select $\overline x.\; $Consequently, the *fl* would be in range: *fl*∈[*fl*_*min*_,*fl*_*max*_]. The opposite number $\overline {fl} $ can be determined by:

6 $$ \overline{fl}=fl_{min}+fl_{max}-x,  $$

Later, evaluate the fitness for the first *fl* value and the fitness for $\overline {fl} $ in all the iterations. When $fitness(fl)\geq fitness(\overline {fl}) $, they select *fl*, or else $\overline {fl} $ would be selected. The stages of presented method can be given in the following.

Step1: The count of crows is *nc*=25,*fl*_*min*_=0.1,*fl*_*max*_=1.8,*AP*=0.3, and the maximal number of iterations is *t*_*max*_=100.

Step2: The position that represent the features are made by *U*(0, 1).

Step3: The fitness function (FF) can be determined by

7 $$ Fitness\;=C+W\times\left(1-\frac{F_{all}}{F_{sub}}\right),\;\;\;\;\;\;\;\;\;\;\;\;\;\;\;\;\;\;\;\;\;\;\;\;\;\;\;\;\;\;\;\;\;\;\;\;\;\;\;\;  $$

Whereas *C* represent the classification performance, *W* represent the weighted factors in the range of zero and one, *F*_*all*_ represent the overall amount of features and *F*_*sub*_ signifies the length of elected feature.

Step4: The position of the crows are upgraded as Eq. (2)

Step5: Steps 3 & 4 are repetitive till a *t*_*max*_ is attained.

### Process involved in OSAE based classification model

At the time of classification process, the chosen features are passed to the OSAE model. AEs have been unsupervised ANN utilized to representation learning. The AE structure has been planned for imposing a bottleneck from the network which forces a compressed knowledge illustration of the input. So, the correlation among the input features is learned and recreated. AEs are encoding-decoding frameworks. The encoding map the original input *x* to hidden layer that has been regarded as latent space representation. The decoding then regenerates this latent representation as to $\widehat x $. The encoder as well as decoder models are determined in Eqs. (8) and (9) correspondingly

8 $$ h=\sigma \left(Wx+b\right),  $$

9 $$ X=\sigma \left(W^{'}h+b^{'}\right),\;  $$

where *x*=(*x*_1_, *x*_2_, …*x*_*n*_) signifies the input data vector, *h*=(*h*_1_, *h*_2_, …*h*_*n*_) refers the low dimension vector reached in the hidden layer, and $\widehat x=(\widehat {x}_{1},\;\widehat {x}_{2},\;\dots \widehat {x}_{n}) $ represents the recreated input. *W* and $\phantom {\dot {i}\!}W^{'}$ defines the weight matrices, *b* and $\phantom {\dot {i}\!}b^{'}$ demonstrated the bias vectors, and *σ* denotes the sigmoid activation function, for instance, $\sigma =\frac {1}{1+e^{-x}} $. It can utilize the MSE function as the recreated error function amongst *h* and X:

10 $$ E=\frac{1}{N}\sum\limits_{i=1}^{N}\left\Vert\widehat{x_{i}}-x_{i}\right\Vert^{2}  $$

**Fig. 2:**
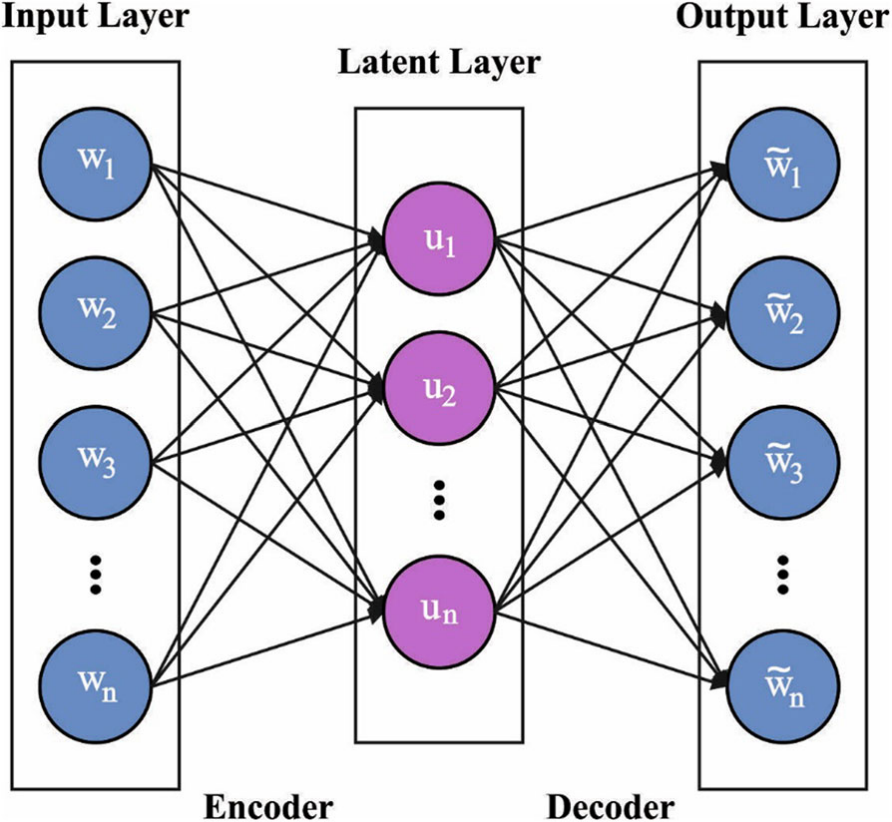
Structure of SAE

Overfitting has been common challenge which occurs if trained AE network. An effectual manner for solving this issue is by implementing a weight penalty to cost function:

11 $$ E=\frac{1}{N} \sum\limits^{N}_{i=1}\frac{1}{2}\left\Vert\widehat{x_{i}}-x_{i}\right\Vert^{2}+\frac{\lambda}{2} \left(\left\Vert W \right\Vert^{2}+\left\Vert W^{'}\right\Vert^{2}\right)  $$

where *λ* refers the weight attenuation coefficients. Moreover, a sparse penalty term has been presented from the AE hidden layer for achieving optimum feature learning in sparse constraint and keep a condition in which the AE copies the input data to outcome [22]. Considering ${\widehat p}_{j} $ represents the average activation of hidden layer neuron, it can be determined as ${\widehat p}_{j}=\frac {1}{N}\Sigma _{i=1}^{N}h_{j}(x_{i}) $, and *ρ* refers the sparsity proportion, frequently a small positive value nearby 0. For achieving sparsity, it can be restricted to ${\widehat \rho }_{j}=\rho $, and the Kullback-Leibler (*KL*) divergence was established for the loss function as regularization term:

12 $$ {}KL(\widehat{\rho}\parallel\rho)={\sum\nolimits}_{j=1}^{K}\rho log\left(\frac{\rho}{\rho_{j}}\right)+\left(1-\rho\right)log\;\left(\frac{1-\rho}{1-\rho_{j}}\right),  $$

where *K* stands for the number of hidden neurons. Therefore, the loss function of sparse AE now has 3 parts: the MSE, weight attenuation, and sparsity regularization parts:

13 $$ {}\begin{aligned} E&=\frac{1}{N}{\sum\nolimits}_{i=1}^{N}\frac{1}{2}\left\| x_{i}-x_{i}\right\|^{2}+\frac{\lambda}{2}\left(\left\| W\right\|^{2}+\left\| W^{'}\right\|^{2}\right)\\ &\quad+\beta KL(\widehat\rho\vert\vert\rho), \end{aligned}  $$

where *β* refers the sparsity regularization parameter. Also, it can stack various sparse AEs for achieving improved feature learning. The framework entails linking the encoder to input layers of the next sparse AE, so make sure the network gains optimum representation learning. Figure 2 depicts the framework of SAE.

During this analysis, the SSAE network is presented. In the SSAE network, the hidden layer of the earlier sparse AE serves as input to the next sparse AE. The last hidden layer is then linked to the oftmax classifier that carries out the classification. So, the presented SSAE network contains train sparse AEs and a Softmax classifier. The BP was implemented for fine-tuning the parameter of the total network with the trained samples and their labels. The fine-tuning stage assumes the many layers of the network as one model. Considering {*y*^1^, *y*^2^, …,*y*^*m*^} implies the target variable of the trained data, the cost function of the whole network is determined as:

14 $$ E=-\frac1m\left[{\sum\nolimits}_{i=1}^{m}{\sum\nolimits}_{j=1}^{N}1\left\{y^{i}=j\right\}\log\;\frac{e^{\theta_{i}^{T}x^{i}}}{\Sigma_{l=1}^{N}e^{\theta_{l}^{T}x^{i}}}\right],\;\;\;\;\;\;\;\;\;\;\;\;\;\;\;\;\;\;\;\;\;\;\;\;\;\;  $$

where 1{∙} indicates the indicator function, for instance, 1{*y*^*i*^=*j*}=1 if *y*=1, and 1{*y*^*i*^=*j*}=0 if *y*≠*j*,*N* implies the number classes, and *θ*_*i*_ represents the weight matrix associating the *ith* output unit. In addition, it can employ the EO technique for optimizing the SSAE parameter, for instance, an optimum weight as well as bias values. The selection of weights as well as bias are vital from trained robust NNs.

The parameter optimization of SAE model takes place using the EO algorithm [23]. It follows dynamic mass balance system that runs on control volume. An arithmetical expression is utilized for representing a mass balance to determine the concentration of a non-reactive constituent under the dynamic environments of control volume. This expression is a function with their several processes under the different kinds of source and sink. The entire description of the EO approach can be explained by the following: The haphazard population (primary concentration) is initiated by standard distribution depending on amount of particles and dimensional in provided search region:

15 $$ C_{i}^{initial}=C_{min}+rand_{i}\left(C_{max}-C_{min}\right)i=1,2,\dots,\;n\;\;\;\;\;\;\;\;\;\;\;\;\;\;\;\;\;\;\;\;\;\;\;\;\;\;\;\;\;\;\;\;\;\;\;\;\;  $$

where as $C_{i}^{initial} $ represent vector of early concentration of *ith* particles, *C*_*min*_ and *C*_*max*_ represents lower and upper bounds, *rand*_*i*_ indicates uniform arbitrary number from the range of zero and one and *n* denotes the size of population.

In order to define the equilibrium state (global optimal), a pool of 4 optimum to this point candidates should be found along with other particles using a concentration equivalent to arithmetical mean of these 4 particles. Thus particles together procedure a pool vector as follows

16 $$ {\overrightarrow C}_{eq.pool}=\left({\overrightarrow C}_{(1)},{\overrightarrow C}_{(2)},{\overrightarrow C}_{(3)},{\overrightarrow C}_{(4)},{\overrightarrow C}_{eq(ave)}\right\}  $$

In the evolution phase, initial particle updates its concentration in initial generation according to on ${\overrightarrow C}_{eq(1)} $ and in next generation, then upgrading might takes place on ${\overrightarrow C}_{eq(ave)} $. Then, all the particles with each candidate solution are upgraded until the completion of evolution process.

The exponential word *F* displayed in Eq. (3) helps EO method by attaining an appropriate balance among intensification and diversification. *λ* represent an arbitrary number from the range zero and one for controlling the turn-over rate in actual control volume.

17 $$ \overrightarrow F=e^{-\overrightarrow\lambda(t-t_{0})}  $$

Whereas *t* represents the amount of iteration (*Iter*) as follows:

18 $$ t=\left(1-\frac{Iter}{Max_{-}iter}\right)\left(a_{2}\frac{Iter}{Max_{-}iter}\right),  $$

In which *Iter*=*current* *iteration*,*Max*_*iter*= maximum iteration and parameters *a*_2_ is used for controlling exploitation capability of EO [24]. For ensuring the convergence when improving local and global search capability of the method:

19 $$ \overset{\rightharpoonup}{t_{0}}=\frac{1}{\overset{\rightharpoonup}{\lambda}}ln\left(-a_{1}sign\left(\overset{\rightharpoonup}{r}-0.5\right)\left(1-e^{-\overset{\rightharpoonup}{\lambda}t}\right]\right)+t,  $$

In which *a*_1_ & *a*_2_ is utilized for controlling global as well as local search capability of EO method. The $sign(\vec {r}-0.5),$ is accountable for the way of exploitation and exploration. In *EO*, the *a*_1_ & *a*_2_ values are selected to be 2 and 1 correspondingly.

By replacing Eq. (19) in Eq. (17), the equation would become:

20 $$ \overset{\rightharpoonup}{F}=a_{1}sign\left(\overset{\rightharpoonup}{r}-0.5\right)\left[e^{-\overset{\rightharpoonup}{\lambda} t}-1\right],  $$

The generation rate in EO is employed for improving exploitation as a function of time. The initial order exponential decay procedure in the method of generation rate of multi-purpose models:

21 $$ \overset{\rightharpoonup}{G}={\overset{\rightharpoonup}G}_{0}e^{-\overset{\rightharpoonup}{k}(t-t_{0})}\;\;,  $$

Whereas *G*_0_= first value, *k*= decay variable.

Lastly, the generation rate expression assumes *k*=*λ*:

22 $$ \overset{\rightharpoonup}{G}={\overset{\rightharpoonup}{G}}_{0}e^{-\overset{\rightharpoonup}{\lambda}(t-t_{0})}={\overset{\rightharpoonup}{G}}_{0}{\overset{\rightharpoonup}{F}}_{0}\;,  $$

23 $$ {\overset{\rightharpoonup}{G}}_{0}=G\overset{\rightharpoonup}{C}P\left({\overset{\rightharpoonup}{C}}_{eq}-\overset{\rightharpoonup}{\lambda}\overset{\rightharpoonup}{C}\right),  $$

24 $$ G\overset{\rightharpoonup}{C}P=\left(\begin{array}{cc} 0.5r_{1},&r_{2}\geq0\\ 0,&r_{2}<0\end{array}\right.,  $$

Let *r*_1_,*r*_2_ be the 2 arbitrary numbers from the range of [0, 1] and *GCP* variable is for controlling generation rate.

Based on the above equation, the last upgrading equation of concentration (particles) is determined by:

25 $$ \overset{\rightharpoonup}{C}={\overset{\rightharpoonup}{C}}_{eq}+\left(\overset{\rightharpoonup}{C}- {\overset{\rightharpoonup}{C}}_{eq}\right)\overset{\rightharpoonup}{F}+\frac{\overset{\rightharpoonup}{G}}{\overset{\rightharpoonup}{\lambda} V}\left(1-\overset{\rightharpoonup}{F}\right),  $$

The upgrading equation has 3 terms: initial term is an equilibrium concentration; the next term is used to global search and last term has been accountable to local search for attaining solution more precisely. In order to optimally adjust the parameter of the SAE algorithm, the EO model is utilized and the thorough functioning can be given as follow. The training method of the SAE algorithm is performed via a FF. Additionally, ten-fold cross-validation procedure is used for evaluating the FF. In ten-fold CV, the training databases are arbitrarily segmented to a set of ten mutually exclusive subsets of almost equivalent size whereas 9 subsets are utilized for training the information and the residual one is employed for testing the information. This processes are repetitive for the collection of ten iterations that the subsets utilize for testing the method. The FF is represented as 1−*CA*_*validation*_of the ten-fold CV method in the training data, as determined in Eq. (26). As well, a solution with maximal *CA*_*validation*_ results in minimum fitness value.

26 $$ Fitness\;=1-CA_{validation},  $$

27 $$ CA_{validation}=1-\frac1{10}{\sum\nolimits}_{i=1}^{10}{\vert\frac{y_{c}}{y_{c}+y_{f}}\vert\times100}\;,  $$

Whereas, *y*_*c*_ & *y*_*f*_ indicates the amount of true and false classification. Lastly, the hyperparameter included in the SAE algorithm is optimally picked up by the EO method in which the classification performances get enhanced.

## Experimental validation

**Fig. 3:**
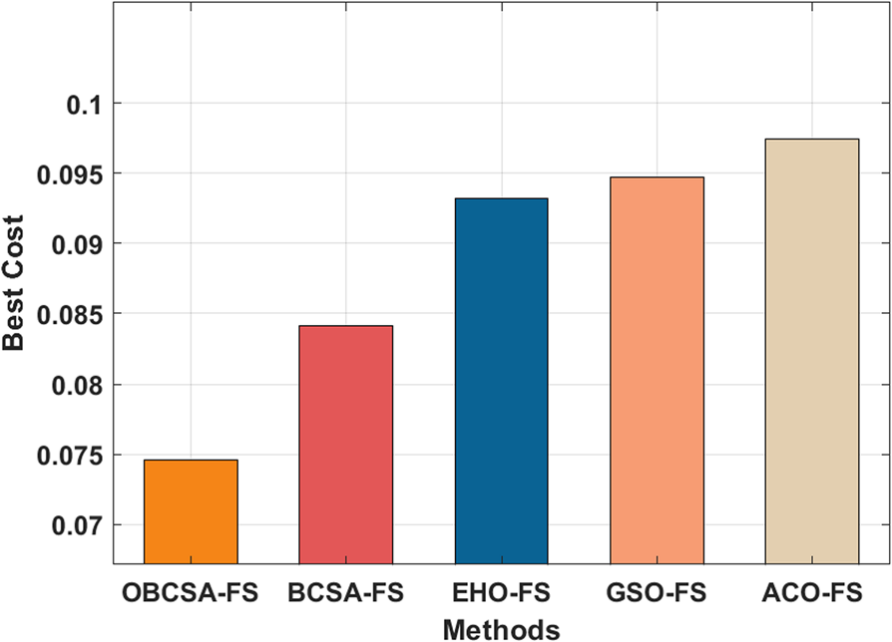
Best cost analysis of OBCSA-FS model

The performance validation of the OBCSA-OSAE model takes place using a data set collected from an obstetrics and gynecology hospital in Hebei province during 2020-2021. It comprises 11000 instances with a total of 149 features. Among the available instances, a set of 1042 samples comes under the presence of PPH. As the trial and error we performed the test and train with variable size to data starting from 50 percent of test and same amount of training data. Generally machine learning to need a huge dat to train the algorithm m results are displayed with The best cost analysis of the OBCSA-FS with existing techniques are demonstrated in Table 1 and Fig. 3. The results demonstrated that the GSO-FS and ACO-FS techniques have showcased ineffective outcomes with the maximum best cost of 0.09468 and 0.09741 respectively. In line with, the EHO-FS and BCSA-FS techniques have obtained a moderate best cost of 0.09321 and 0.08412 respectively. But the OBCSA-FS technique has accomplished better outcomes with the least best cost of 0.07462.

**Table 1:** Best cost analysis of OBCSA-FS model

Methods	No. of Selected Features	Best Cost
OBCSA-FS	15	0.07462
BCSA-FS	24	0.08412
EHO-FS	36	0.09321
GSO-FS	40	0.09468
ACO-FS	48	0.09741

**Fig. 4:**
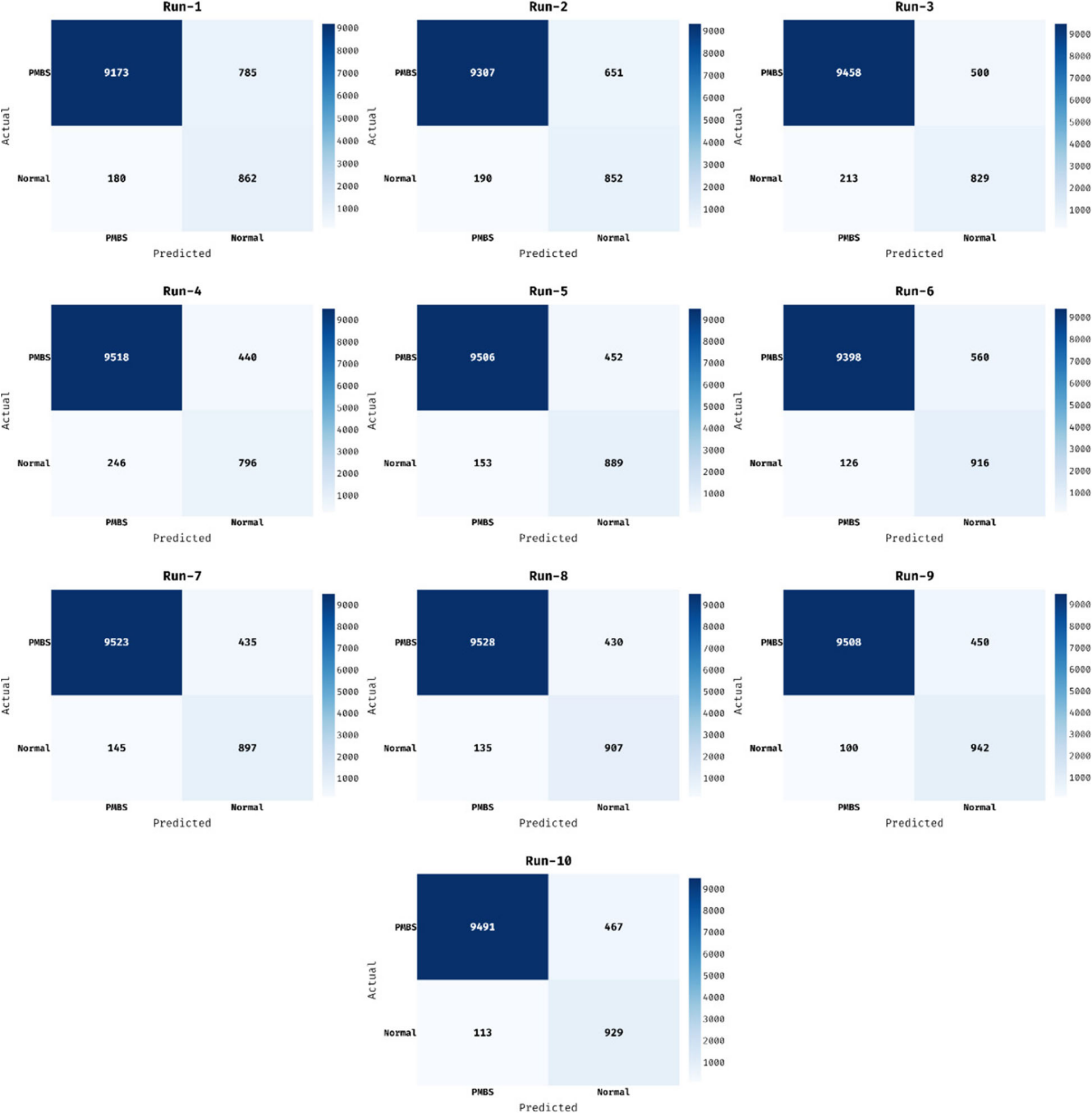
Confusion matrix of OBCSA-OSAE model with distinct runs

The confusion matrix produced by the OBCSA-OSAE technique on the execution of ten dissimilar runs is illustrated in Fig. 4. On the test run-1, the OBCSA-OSAE technique has classified 9713 and 862 into PMBS and Normal classes respectively. Also, on the test run-3, the OBCSA-OSAE method has classified 9458 and 829 into PMBS and Normal classes correspondingly. Besides, on the test run-6, the OBCSA-OSAE algorithm has classified 9398 and 916 into PMBS and Normal classes respectively. At the same time, on the test run-8, the OBCSA-OSAE approach has classified 9508 and 942 into PMBS and Normal classes respectively. Eventually, on the test run-10, the OBCSA-OSAE system has classified 9491 and 929 into PMBS and Normal classes correspondingly.

**Fig. 5:**
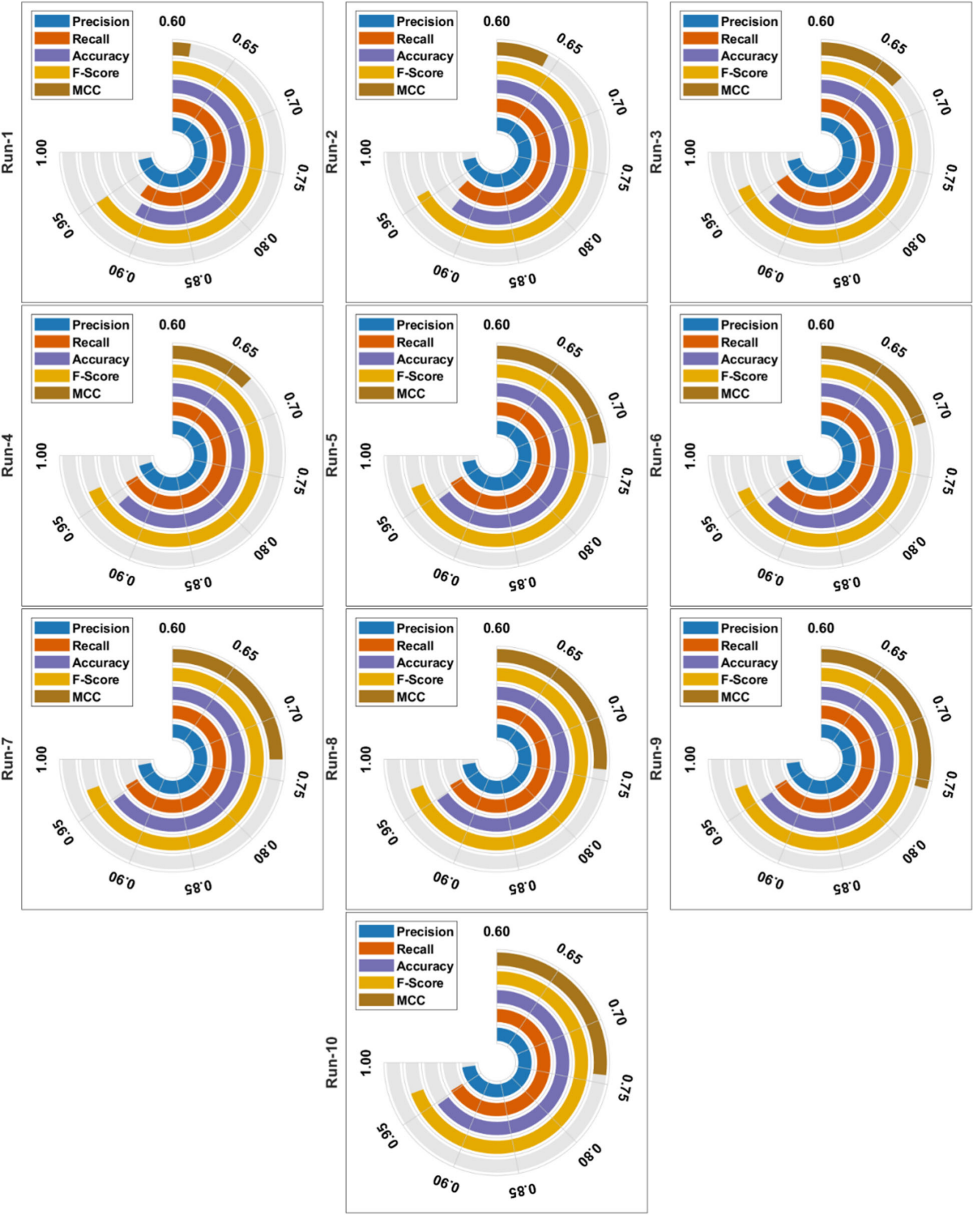
Result analysis of OBCSA-OSAE model with different runs

Table 2 and Fig. 5 tabulates the overall classification results analysis of the OBCSA-OSAE technique under ten runs. The OBCSA-OSAE technique has gained effectual outcomes under every run.

**Table 2:** Result analysis of OBCSA-OSAE model with different measures

No. of Runs	Precision	Recall	Accuracy	F-Score	MCC	Error Rate
Run-1	0.9808	0.9212	0.9123	0.9500	0.6143	0.0877
Run-2	0.9800	0.9346	0.9235	0.9568	0.6414	0.0765
Run-3	0.9780	0.9498	0.9352	0.9637	0.6697	0.0648
Run-4	0.9748	0.9558	0.9376	0.9652	0.6674	0.0624
Run-5	0.9842	0.9546	0.9450	0.9692	0.7230	0.0550
Run-6	0.9868	0.9438	0.9376	0.9648	0.7069	0.0624
Run-7	0.9850	0.9563	0.9473	0.9704	0.7335	0.0527
Run-8	0.9860	0.9568	0.9486	0.9712	0.7414	0.0514
Run-9	0.9896	0.9548	0.9500	0.9719	0.7565	0.0500
Run-10	0.9882	0.9531	0.9473	0.9704	0.7431	0.0527
Average	0.9833	0.9481	0.9384	0.9654	0.6997	0.0616

## Result analysis of OBCSA-OSAE model with different runs

With run-1, the OBCSA-OSAE technique has obtained precision, recall, accuracy, F-score, MCC, and error rate of 0.9808, 0.9212, 0.9123, 0.9500, 0.6143, and 0.0877. Similarly, with run-3, the OBCSA-OSAE methodology has attained precision, recall, accuracy, F-score, MCC, and error rate of 0.9780, 0.9498, 0.9352, 0.9637, 0.6697, and 0.0648. Followed by, in run-6, the OBCSA-OSAE approach has obtained precision, recall, accuracy, F-score, MCC, and error rate of 0.9868, 0.9438, 0.9376, 0.9648, 0.7069, and 0.0624. Finally, with run-10, the OBCSA-OSAE approach has obtained precision, recall, accuracy, F-score, MCC, and error rate of 0.9882, 0.9531, 0.9473, 0.9704, 0.7431, and 0.0527.

**Fig. 6:**
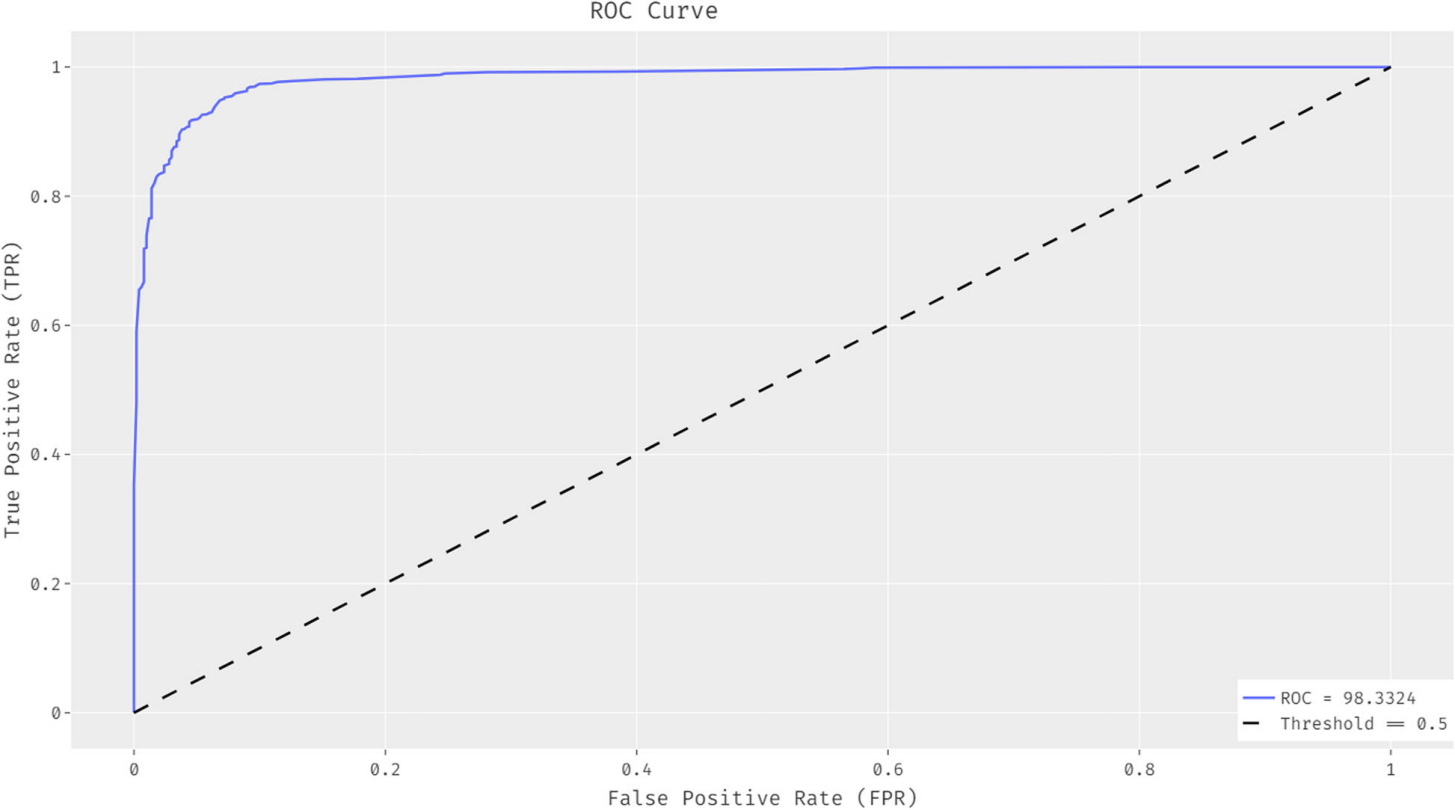
ROC analysis of OBCSA-OSAE model

The ROC analysis of the OBCSA-OSAE technique is offered in Fig. 6. The figure shows that the OBCSA-OSAE technique has accomplished effectual outcomes with a higher ROC of 98.3324%.

**Fig. 7:**
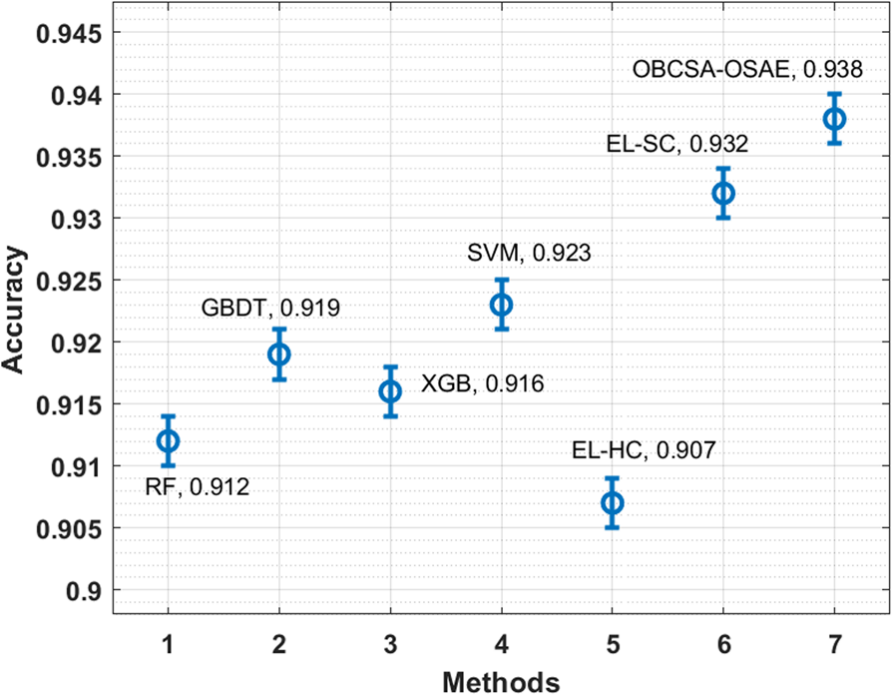
Accuracy analysis of OBCSA-OSAE model with existing techniques

Finally, a brief comparative result analysis of the OBCSA-OSAE technique with existing ones is made in Table 3 [25]. The accuracy analysis of the OBCSA-OSAE technique with compared methods is provided in Fig. 7. The figure displays that the EL-HC, RF, XGB, and GBDT techniques have moderately closer accuracy values. At the same time, the SVM and EL-SC techniques have obtained slightly improved accuracy values. However, the OBCSA-OSAE technique has outperformed the other techniques with a maximum accuracy of 0.938.

**Table 3:** Comparative analysis of OBCSA-OSAE model with existing methods in terms of different measures

Methods	Accuracy	Recall	Precision	F-Score	MCC
RF	0.912	0.747	0.937	0.830	0.630
GBDT	0.919	0.655	0.736	0.690	0.670
XGB	0.916	0.739	0.878	0.800	0.660
SVM	0.923	0.748	0.937	0.830	0.640
EL-HC	0.907	0.656	0.953	0.780	0.650
EL-SC	0.932	0.697	0.847	0.760	0.690
OBCSA-OSAE	0.938	0.948	0.983	0.965	0.700

**Fig. 8:**
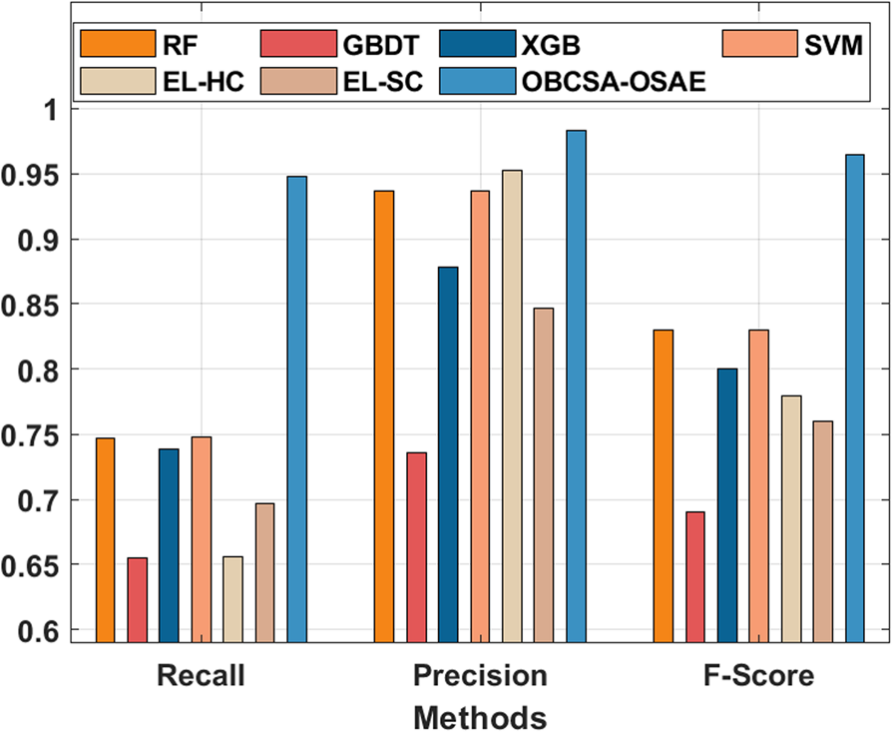
Comparative analysis of OBCSA-OSAE model with different measures

The recall, precision, and F-score analysis of the OBCSA-OSAE approach with related techniques are given in Fig. 8. The figure demonstrated that the EL-HC, RF, XGB, and GBDT methods have outperformed moderately closer recall, precision, and F-score values. In addition, the SVM and EL-SC methods have reached somewhat enhanced recall, precision, and F-score values. Finally, the OBCSA-OSAE methodology has exhibited the other systems with higher recall, precision, and F-score of 0.948, 0.983, and 0.965.

**Fig. 9:**
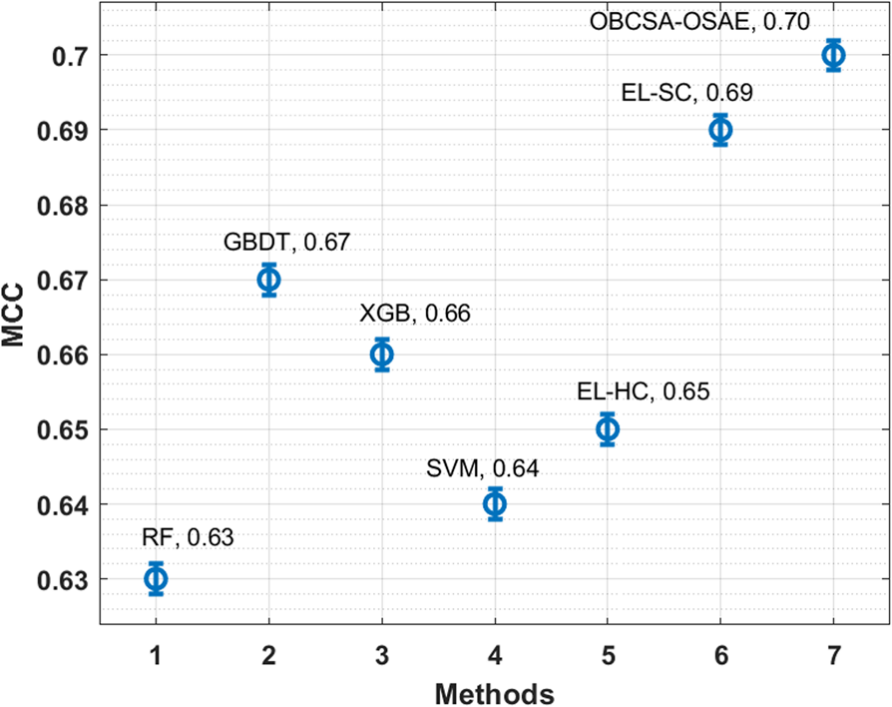
MCC analysis of OBCSA-OSAE model with existing approaches

The MCC analysis of the OBCSA-OSAE technique with compared methods is provided in Fig. 9. The figure depicts that the EL-HC, RF, XGB, and GBDT manners have moderately closer MCC values. Simultaneously, the SVM and EL-SC approaches have somewhat superior MCC values. Lastly, the OBCSA-OSAE algorithm show the other techniques with the maximal MCC of 0.700. From these result analysis, the OBCSA-OSAE technique has accomplished maximum PPH prediction outcomes.

## Conclusion

In this study, an effective OBCSA-OSAE technique was derived for the detection and classification of PPH. The proposed OBCSA-OSAE technique encompassed two major stages, namely selection of features and classification. Primarily, the OBCSA technique was utilized to optimally select a subset of features. Then, the EO algorithm served to optimally choose the parameters involved in the SAE model. Finally, the SAE model was used for the classification of PPH. For showcasing the improved performance of the OBCSA-OSAE approach was varied out on the benchmark data set. The experimental outcomes highlighted that the OBCSA-OSAE technique has demonstrated the other techniques featured in terms of different evaluation parameters. In future research, the clustering process could be included to handle massive amounts of PPH data and improve classification outcomes.In the future, we would like to analyze on the time complexity of the algorithm comparing the various parameters.

## Data Availability

Data used to support the findings of this study are available from the corresponding author upon request.

## References

[1] World Health Organization (2012). WHO Recommendations for the Prevention and Treatment of Postpartum Haemorrhage.

[2] Callaghan WM, Kuklina EV, Berg CJ (2010). Trends in postpartum hemorrhage: the United States, 1994–2006. Am J Obstet Gynecol.

[3] Einerson BD, Miller ES, Grobman WA (2015). Does a postpartum hemorrhage patient safety program result in sustained changes in management and outcomes?. Am J Obstet Gynecol.

[4] Main EK, Goffman D, Scavone BM (2015). National partnership for maternal safety: consensus bundle on obstetric hemorrhage. Obstet Gynecol.

[5] Chen C, Liu X, Chen D, Huang S, Yan X, Liu H, Chang Q, Liang Z (2019). A risk model to predict severe postpartum hemorrhage in patients with placenta previa: A single-center retrospective study. Ann Palliat Med.

[6] Koopmans CM, Tuuk KVD, Groen H, Doornbos JPR, Graaf IMD (2014). Prediction of postpartum hemorrhage in women with gestational hypertension or mild preeclampsia at term. Acta Obstet Gynecol Scand.

[7] Kramer MS, Berg C, Abenhaim H, Dahhou M, Rouleau J, Mehrabadi A, Joseph KS (2013). Incidence, risk factors, and temporal trends in severe postpartum hemorrhage. Amer J Obstet Gynecol.

[8] Onan A (2015). On the performance of ensemble learning for automated diagnosis of breast cancer. Adv Intell Syst Comput.

[9] Sidey-Gibbons JAM, Sidey-Gibbons CJ (2019). ‘Machine learning in medicine: A practical introduction. BMC Med Res Methodol.

[10] Mienye ID, Sun Y, Wang Z (2020). An improved ensemble learning approach for the prediction of heart disease risk. Informat Med Unlocked.

[11] Venkatesh KK, Strauss RA, Grotegut C, Heine RP, Chescheir NC, Stringer JS, Stamilio DM, Menard MK, Jelovsek JE (2020). Machine learning and statistical models to predict postpartum hemorrhage. Obstet Gynecol.

[12] Kumar VA, Sharmila S, Kumar A, Bashir AK, Rashid M, Gupta SK, Alnumay WS (2021). A novel solution for finding postpartum hemorrhage using fuzzy neural techniques. Neural Computing and Applications.

[13] Wu Q, Yao K, Liu Z, Li L, Zhao X, Wang S, Shang H, Lin Y, Wen Z, Zhang X, Tian J (2019). Radiomics analysis of placenta on T2WI facilitates prediction of postpartum hemorrhage: a multicenter study. EBioMedicine.

[14] Betts KS, Kisely S, Alati R (2019). Predicting common maternal postpartum complications: Leveraging health administrative data and machine learning. BJOG Int J Obstet Gynaecol.

[15] Man Z. Comparative Study of Machine Learning Models to Predict PPH. Master’s Paper. The University of North Carolina at Chapel Hill; 2019.

[16] Kumar VD, Sharmila S, Kumar A, Subha SS, Singh V, Kannan R (2020). Predictive Analysis of Postpartum Haemorrhage And Hypothermia Using Wearable Device. Eur J Mol Clin Med.

[17] Hochman E, Feldman B, Weizman A, Krivoy A, Gur S, Barzilay E, Gabay H, Levy J, Levinkron O, Lawrence G (2021). Development and validation of a machine learning-based postpartum depression prediction model: A nationwide cohort study. Depression Anxiety.

[18] Yang J, Guo P, Song Y, Han L, Yang X, Bai H. A Clinical Decision Support System for Prediction of Postpartum Hemorrhage in Vaginal Birth. 2021. 10.21203/rs.3.rs-618703/v1.

[19] Zheutlin AB, Vieira L, Shewcraft RA, Li S, Wang Z, Schadt E, Gross S, Dolan SM, Stone J, Schadt E, Li L (2022). Improving postpartum hemorrhage risk prediction using longitudinal electronic medical records. Journal of the American Medical Informatics Association: JAMIA.

[20] Askarzadeh A (2016). A novel metaheuristic method for solving constrained engineering optimization problems: crow search algorithm. Comput Struct.

[21] Al-Thanoon NA, Algamal ZY, Qasim OS (2021). Feature selection based on a crow search algorithm for big data classification. Chemometrics and Intelligent Laboratory Systems.

[22] Mienye ID, Sun Y (2021). Improved Heart Disease Prediction Using Particle Swarm Optimization Based Stacked Sparse Autoencoder. Electronics.

[23] Faramarzi A, Heidarinejad M, Stephens B, Mirjalili S (2020). Equilibrium optimizer: A novel optimization algorithm. Knowl-Based Syst.

[24] Dinkar SK, Deep K, Mirjalili S, Thapliyal S (2021). Opposition-based laplacian equilibrium optimizer with application in image segmentation using multilevel thresholding. Expert Syst Appl.

[25] Zhang Y, Wang X, Han N, Zhao R (2021). Ensemble Learning Based Postpartum Hemorrhage Diagnosis for 5G Remote Healthcare. IEEE Access.

